# ncRNAs and polyphenols: new therapeutic strategies for hypertension

**DOI:** 10.1080/15476286.2022.2066335

**Published:** 2022-04-19

**Authors:** Elham Shirazi-Tehrani, Alireza Chamasemani, Negar Firouzabadi, Marzieh Mousaei

**Affiliations:** a Department of Pharmacology & Toxicology, School of Pharmacy, Shiraz University of Medical Sciences, Shiraz, Iran; b Department of Cardiology, School of Medicine, Isfahan University of Medical Sciences, Isfahan, Iran; c Archaea Centre, Department of Biology, University of Copenhagen, Copenhagen N, Denmark

**Keywords:** Polyphenol, ncRNA, microRNA, long non-coding RNA, blood pressure, hypertension

## Abstract

Polyphenols have gained significant attention in protecting several chronic diseases, such as cardiovascular diseases (CVDs). Accumulating evidence indicates that polyphenols have potential protective roles for various CVDs. Hypertension (HTN) is among the hazardous CVDs accounting for nearly 8.5 million deaths worldwide. HTN is a complex and multifactorial disease and a combination of genetic susceptibility and environmental factors play major roles in its development. However, the underlying regulatory mechanisms are still elusive. Polyphenols have shown to cause favourable and beneficial effects in the management of HTN. Noncoding RNAs (ncRNAs) as influential mediators in modulating the biological properties of polyphenols, have shown significant footprints in CVDs. ncRNAs control basic functions in virtually all cell types relevant to the cardiovascular system and, thus, a direct link with blood pressure (BP) regulation is highly probable. Recent evidence suggests that a number of ncRNAs, including main small ncRNAs, microRNAs (miRNAs) and long ncRNAs (lncRNAs), play crucial roles with respect to the antihypertensive effects of polyphenols. Indeed, targeting lncRNAs by polyphenols will be a novel and promising strategy in the management of HTN. Herein, we reviewed the effects of polyphenols in HTN. Additionally, we emphasized on the potential effects of polyphenols on regulations of main ncRNAs, which imply the role of polyphenols in regulating ncRNAs in order to exert protective effects and thus proposing them as new targets for HTN treatment.

**Abbreviations :** CVD: cardiovascular disease; BP: blood pressure; HTN: hypertension, lncRNAs: long noncoding RNAs; p38-MAPK: p38-mitogenactivated protein kinase; OPCs: oligomeric procyanidins; GTP: guanosine triphosphate; ROS: reactive oxygen species; cGMP: cyclic guanosine monophosphate; SGC: soluble guanylate cyclase; PI3K: phosphatidylinositol 3-kinase; cGMP: Cyclic GMP; eNOS: endothelial NO synthase; ERK ½: extracellular signal-regulated kinase ½; L-Arg: L-Arginine; MAPK: mitogen-activated protein kinases; NO: Nitric oxide; P: Phosphorus; PDK1: Phosphoinositide-dependent kinase 1; PI3-K: Phosphatidylinositol 3-kinase; PIP2: Phosphatidylinositol diphosphate; ncRNAs: non-protein-coding RNA; miRNAs: microRNAs; OPCs: oligomeric procyanidins; RES: resveratrol; GE: grape extract; T2DM: type 2 diabetes mellitus; IL: interleukin; TNF-α: tumour necrosis factor-alpha; NF-κB: nuclear factor NF-kappa-B; ALP: alkaline phosphatase; PARP1: poly [ADP-ribose] polymerase 1; HIF1a: Hypoxia-inducible-factor 1A; NFATc2: nuclear factor of activated T cells 2; PAD: peripheral artery disease; SHR: spontaneously hypertensive rat; RAAS: renin-angiotensin-aldosterone system; AT_1_R: angiotensin type-1 receptor; Nox: NADPH oxidase; HO-1: haem oxygenase-1; JAK/STAT: Janus kinase/signal transducers/activators of the transcription; PNS: panax notoginseng saponin; snoRNA: small nucleolar RNA; hnRNA: heterogeneous nuclear RNA; VSMCs: vascular smooth muscle cells; irf7: interferon regulatory factor 7; limo2: LIM only domain 2; GWAS: genome-wide association study; GAS5: Growth arrest-specific 5; Asb3, Ankyrin repeat and SPCS box containing 3; Chac2: cation transport regulator homolog 2; Pex11b: peroxisomal membrane 11B; Sp5: Sp5 transcription factor; EGCG: epigallocatechin gallate; ApoE: Apo lipoprotein E; ERK-MAP kinase: extracellular signal-regulated kinases-mitogen-activated protein kinase; PAH: pulmonary artery hypertension; PAP: pulmonary arterial pressure; HIF1a: hypoxia-inducible-factor 1A; NFATc2: nuclear factor of activated T cells 2; HMEC-1: Human microvascular endothelial cells; stat2: signal transducers and activators of transcription 2; JNK: c-Jun N-terminal kinase; iNOS: inducible NO synthase. SNP: single nucleotide polymorphism; CAD: coronary artery disease

## 1. Introduction

Hypertension (HTN) is one of the most common predominant non-communicable medical conditions involving the cardiovascular system and affecting as many as one billion people worldwide with high morbidity and mortality [1]. HTN is a complex and multifactorial illness attributed to both genetic and/or environmental factors involving persistent elevation of systemic blood pressure (BP) [2]. Despite a large degree of available therapeutic options, the prevalence of HTN is predicted to be increased over the next decade [3].

In this context, current evidence strongly suggests that dietary patterns significantly attribute to the management of HTN [4]. Recently, beneficial effects of polyphenols on HTN has gained much attention due to their little side effects and low toxicities [5]. Polyphenol biomolecules (phenolic compounds) are the most widely distributed secondary metabolites from plants in dietary sources with significant antioxidant and anti-inflammatory effects both in vitro and in vivo [6].

A vast body of literature evinces their beneficial effects on human health, especially in diets associated with high consumption of fruits and vegetables [7]. Growing evidence indicates that polyphenols have multiple targets and can mediate multiple cellular signalling pathways in order to exert their antihypertensive effects [8].

Genetic mutations and abnormal synthesis of transcriptional factors play pivotal role in the pathogenesis of HTN [9]. Non-protein-coding RNAs (ncRNAs) are among the small molecules that have increasingly gained attention in various cellular processes. ncRNA is a functional RNA molecule that is transcribed from DNA, but is not translated into proteins and has been shown to be involved in regulating gene expression and inhibiting the translation and degradation of messenger RNAs (mRNAs) [10].

Cellular fate is determined through different expressions of key pathways that are regulated by long non-coding RNAs (lncRNAs). lncRNAs are considered as a member of the ncRNA family. Manipulation of lncRNAs by pathogens or genomic re-arrangements have been shown to exert great effects on disease outcomes [11]. Association of ncRNAs with various human diseases has recently been highlighted, focusing mainly on miRNAs. However, reports on lncRNAs in relation to HTN are limited at present.

After the discovery of ncRNAs, numerous studies have identified that many polyphenols exert their antihypertensive and ant inflammatory effects by regulating different ncRNAs, which are implicated in regulating the progress of various cardiovascular diseases (CVDs) [12].

In this review, we summarize the antihypertensive properties and potential applications of some promising dietary polyphenols, as novel therapeutic strategies for HTN, focusing mainly on ncRNAs.

## 2. Polyphenols and HTN

### 2.1. Classifications of polyphenols

**Figure 1.**
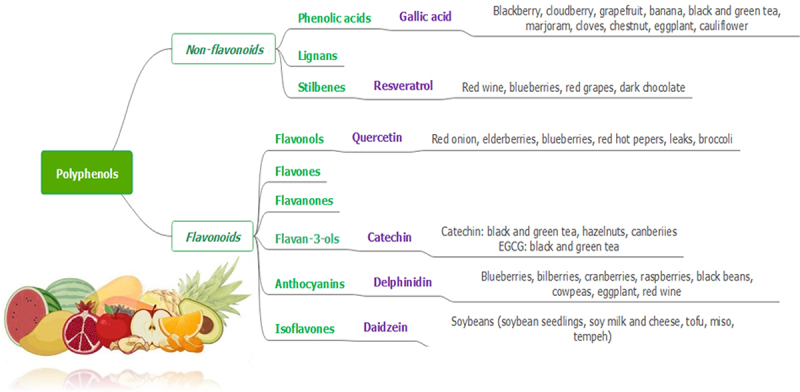
Schematic classification of polyphenols and their natural sources.

Polyphenols are a distinct type of secondary metabolites derived from plants which have multiple potential applications against a number of diseases. Polyphenols are either classified into flavonoids and non-flavonoids, or are subdivided into sub-classes depending on the number of phenolic units within their molecular structure, substituent groups, and/or a linkage type between phenol units. Classifications and natural sources of polyphenols are summarized in Fig. 1.

Polyphenols are classified in to two groups of non-flavonoids and flavonoids. Non-flavonoids are subdivided into phenolic acids, lignans and stilbenes. Flavonoids are subdivided into flavonols, flavones, flavanones, flavan-3-ols, anthocyanins and isoflavones.

The structural diversity of flavonoid molecules rises from variations in hydroxylation pattern and oxidation state resulting in a wide range of compounds such as flavanols, anthocyanidins, isoflavones, flavones, flavonols, flavanones, and flavanonols [13]. Although polyphenols contain several hydroxyl groups located on the aromatic rings, non-flavonoids encompass a single aromatic ring. Non-flavonoid compounds include phenolic acids, stilbenes, and lignans [14].

### 2.2. Biological roles of polyphenols in HTN

**Figure 2.**
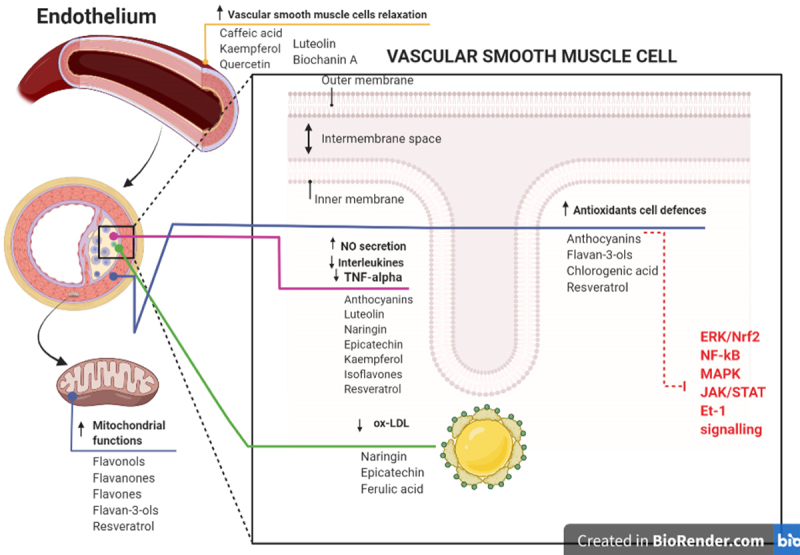
Summary of the potential mechanisms of action through which polyphenols may affect endothelial health and reduce the risk of hypertension.

Recent studies regarding polyphenols primarily encompasses epidemiologic cohort and case-control studies. They have represented that daily intake of polyphenols has a strong impact on the prevention of different types of CVDs including HTN [15]. Besides, numerous studies suggest that polyphenols could exert their antihypertensive and ant inflammatory effects by regulating multiple cellular signalling pathways [16]. The potential mechanisms of action through which polyphenols may exert their protective properties leading to reduced risk of HTN are shown in Fig. 2.

Red note refers to the signalling pathways regulated by polyphenols in hypertension.↑denotes increase, ↓denotes decrease.

#### 2.2.1 Antioxidant properties

Oxidative stress following the production of free radicals and reactive oxygen species (ROS) is a keystone process in various diseases including CVDs [17]. Polyphenols have been shown to counteract the process of oxidative stress and play as strong antioxidants neutralizing the produced free radicals [18]. Accumulating evidence indicates that flavonoids have potential antioxidant properties. There is an emerging view that flavonoids and their metabolites may not only act as conventional hydrogen-donating antioxidants but may also exert modulatory actions in cells by targeting protein and lipid kinases signalling pathways [19].

The most common mechanism of action of flavonoids in modulation of BP is their antioxidant properties. It has been documented that these polyphenols are potent direct scavengers of free radicals and ROS [20]. Flavonoids are oxidized by free radicals, resulting in more stable and less reactive compounds; they have been used as reducing agents as well. Among the most potent antioxidant compounds, flavonols (i.e. quercetin), flavanones (i.e. naringenin and hesperidin), flavones (i.e. apigenin), flavan-3-ols (i.e. catechins), stilbenes (i.e. resveratrol), have been shown to directly scavenge free radicals and thus, restore vascular functionality [21]. Dietary pro-anthocyanidins exhibit characteristics of both antioxidants and signalling molecules and their antioxidant properties is attributed to scavenging ROS [22].

Moreover, polyphenols by providing antioxidant effects, may counteract senescence and thereby restore the mitochondrial function of vascular smooth muscle and endothelial cells by modulation of signal transduction [4]. For instance, flavonoid subgroups such as flavanones and anthocyanins, as well as phenolic acids such as caffeic acid, have shown antioxidant activities by enhancing cellular defence through activation of transcription factors of antioxidant and cytoprotective enzymes, such as the extracellular signal-regulated kinase (ERK)/nuclear factor (erythroid-derived 2)-like 2 (Nrf2) signalling pathway [23].

It is noteworthy that polyphenols also modulate different signalling pathways which may lead to upregulation of key anti-oxidative genes, such as haem oxygenase-1 (HO-1), NAD(P)H dehydrogenase quinone 1 (NQO1), glutamate–cysteine ligase (through its catalytic subunit–GCLC), and induction of endogenous antioxidant enzymes, such as glutathione peroxidase (GPX), superoxide dismutase (SOD), catalase and glutathione reductase (GR), which are substantial elements in the process of oxidative stress [24].

Among the pathways being over activated in CVDs and HTN, is the renin-angiotensin-aldosterone system (RAAS). Activation of RAAS and as a consequence, increased circulating and tissue levels of its primary effector peptide, angiotensin II (Ang II), are among the most prominent processes observed in HTN. Alongside, oxidative stress markers were shown to be elevated and correlated with circulating AngII levels in HF patients. Ang II-mediated hypertrophy was observed to be attenuated by delphinidin (an anthocyanidin), which was reflective of reduced activation of extracellular signal-regulated kinases 1/2) (ERK_1/2_), mitogen-activated protein kinases (MAPK) and c-Jun N-terminal kinase (JNK). Additionally, reports indicate that pretreatment with delphinidin, significantly reduced both H_2_O_2_ and O_2_^•−^, which was reflective of reduced NADPH oxidase (Nox) activity, particularly Nox_2_ [25].

The new venue in the treatment of CVDs has focused on the application of antioxidants and free radical scavenging molecules in order to target the ROS produced as the consequence of overproduction of Ang II. Antioxidant therapy may have an advantage over conventional therapies, such as angiotensin converting enzyme inhibitors (ACEIs) and angiotensin receptor blockers (ARBs), due to their ability to scavenge the ROS generated not only by Ang II, but also by the pro-inflammatory cytokines over-produced in CVDs [25] such as HTN.

#### 2.2.2 Anti-Inflammatory action

Chronic inflammation plays a central role in numerous non-communicable diseases, including CVDs and HTN. Major inflammatory biomarkers in this process include various cytokines such as Interleukin (IL) 1, 3, 6, 8, and 18, tumour necrosis factor-alpha (TNF-α), and macrophage colony-stimulating factor. Phenolic compounds exert anti-inflammatory activities by altering the recruitment of inflammatory cells, decreasing the production of pro-inflammatory molecules such as TNF-α, IL-6 and C-reactive protein (CRP), and inhibiting the production of adhesion molecules such as vascular cell adhesion molecule-1 (VCAM-1) and intercellular adhesion molecule-1 (ICAM-1)] by the endothelium, thereby suppressing cellular migration of monocytes into the sub-endothelial space [26]. Polyphenols are likely to promote their anti-inflammatory properties by modulating transcriptional pathways and/or signalling cascades that modulate gene expression leading to inhibition of inflammatory mediators. Among the polyphenols, some flavonoids such as anthocyanins and flavan-3-ols, phenolic acids (including chlorogenic and caffeic acids), and resveratrol are the most promising anti-inflammatory agents due to affecting the aforementioned inflammatory biomarkers and through regulation of several signalling pathways, including MAPK, janus kinase/signal transducers/activators of the transcription (JAK/STAT), and the NF-κB pathways [27,28]. Strong correlation between dyslipidemia and HTN is well documented. Concerning vascular health, migration and accumulation of oxidized LDL cholesterol (ox-LDL cholesterol), which is formed as a result of ROS and free radical attack in vascular intima, has been long considered as one of the main events in CVDs. However, it only represents the first step in the development of the disease, which also involves macrophages’ activity in discharging various mediators of inflammation, sustaining the whole process of infiltration of smooth muscle cells, formation of foam cells, and infiltration/proliferation of leukocytes [29]. Many polyphenols have been reported to bind to LDL by various mechanisms, providing a protection in CDVs such as HTN [30,31].

#### 2.2.3 Maintenance of endothelial health

Vascular functions, including maintenance of vascular tone, redox balance, and inhibition of platelet aggregation and coagulation, are key factors for endothelial health and prevention of HTN and atherosclerosis [32].

Endothelial cells produce substances needed for the maintenance of healthy vascular function, including nitric oxide (NO), carbon monoxide (CO), endothelium-dependent hyperpolarizing factors, endothelium-derived contracting factors, vasoactive prostanoids and prostacyclin, endothelin (ET), and superoxide [33].

Endothelial dysfunction is markedly evoked by reduced availability of NO as a consequence of increased oxidative stress, generation of free radicals, and other stress factors. Polyphenols may improve the release of NO from the endothelial cells, leading to activation of cyclic guanosine monophosphate (cGMP) in vascular smooth muscle cells and exert blood vessel relaxation, as well as antioxidant, anti-inflammatory, and antithrombotic effects [34].

Flavonoids, such as anthocyanins, flavones (i.e. luteolin), flavanones (i.e. naringin), flavan-3-ols (i.e. epicatechin), flavonols (i.e. kaempferol), isoflavones, and resveratrol may play a direct role in improving the bioavailability of NO in the bloodstream by augmenting the activation of inducible NO synthase (iNOS) and endothelial NO synthase (eNOS) provided by modulation of signalling pathways; for instance, through alteration in phosphatidylinositol 3-kinase (PI3K)/Akt or the adenosine monophosphate-activated protein kinase (AMPK) pathways [35].

Majority of polyphenols such as caffeic acid, kaempferol, quercetin, luteolin, and biochanin A, may exert vasorelaxing effects also by directly acting on vascular smooth muscle cells (through activation of BK channels or inhibition of Ca2+ channels) or indirectly (through activation of Ca2+-activated K+ channels in endothelial cells, leading to hyperpolarization and inhibition of Ca2+ influx into vascular smooth muscle cells), leading to vasorelaxation [36]. However, some polyphenols, such as resveratrol, have been shown to act through more than one of the aforementioned mechanisms [37].

Resveratrol [38], a vasoactive polyphenol, is abundantly found in red wine and has the ability to activate eNOS by stimulating the membrane oestrogen receptor. Some of these beneficial effects may in part be due to resveratrol being a phytoestrogen [39].

#### 2.2.4 Modulating platelet aggregation

Notoginsenoside Ft-1 (Ft-1), a saponin from Panax notoginseng (PNS), can enhance platelet aggregation by activating signalling network mediated through P2Y12 (a Gq-coupled receptor that initiates ADP-induced platelet aggregation) and induce proliferation, migration, and tube formation in cultured human umbilical vein endothelial cells. Reports indicate that Ft-1 enhances proliferation of fibroblasts via activating PI3K/Akt/mTOR signalling pathway. Moreover, Ft-1 has shown to accelerate wound healing by orchestrating multifaceted factors in promoting re-epithelialization, granulation tissue formation, synthesis of collagen, angiogenesis, and preventing excessive inflammatory responses [25]. Several polyphenol groups, such as apigenin, curcumin and luteolin, quercetin, epicatechin, and resveratrol, have been shown to reduce platelet aggregation through the aforementioned anti-inflammatory pathways and also by inhibition of adhesion molecules [40,41].

The effects of polyphenolic compounds and their molecular actions in the cardiovascular system depends on both the type of the polyphenol and the respective CVD. Recent findings are suggestive of the interplay of polyphenols and transcriptional regulators mainly lncRNAs [9] which are thoroughly discussed in the next sections

## 3. A brief history and applications of ncRNAs

Since the 1950s, various types of ncRNAs and the main RNA participants in gene expression such as the ribosomal RNAs (rRNAs), transfer RNAs (tRNAs), messenger RNA (mRNA), small nucleolar RNA (snoRNA), small nuclear RNA (snRNA), micro RNA (miRNA), the RNA component of the signal recognition particle (7SL RNA), lncRNAs, circular RNA, and heterogeneous nuclear RNA (hnRNA) have been discovered and their central roles as scions of protein synthesis have been greatly established [42]. ncRNAs play central roles in the regulation of gene expression, from transcription to splicing and translation. They also contribute significantly to genome organization and stability.

Based on their sizes, ncRNAs are categorized in to three groups: small, about 20 nucleotides; intermediate, less than 200 nucleotides, and longer than 200 nucleotides [43].

Small ncRNAs are categorized in to small interfering RNAs (siRNAs) and miRNAs. Intermediate ncRNAs are small nuclear RNAs that are involved in splicing during protein synthesis, nucleolar RNAs that modify ribosome RNA, transcription start site (TSS)-associated RNAs and promoter-associated small RNAs [44]. Other ncRNAs with sizes over 200 nucleotides are categorized as lncRNAs.

These small, single-stranded RNA sequences not only help us improve our understanding of the physiological and pathophysiological processes at the cellular level, but might also become a valuable part of clinical practice in the future [45].

Eiring et al. reported that the dual role of miRNA genes in silencing activity is through base pairing with mRNA targets or decoying activity that interferes with the function of regulatory proteins in the development of different diseases. By the same token, the authors introduced a new concept that miRNAs can work as molecular decoys for RNA-binding proteins [46].

In the miRNAs biogenesis pathway, Drosha and Dicer, the two miRNA processing enzymes that are required for the maturation of miRNAs, are spatially separated, being localized in the nucleus and in the cytoplasm, respectively. Main function of miRNAsis to inhibit protein synthesis of protein-coding genes, either by inhibition of translation or mRNA degradation. Additionally, miRNAs can switch between translation repression and activation in coordination with cell cycle [47].

miRNAs which are most abundant in the skeletal or heart muscle, are the so-called myomiRs [48]. miR-1, miR-133, miR-208 and miR-499 are among the most widely investigated miRNAs in cardiac pathologies [49,50].

A recent report suggested that miR-1-1 and miR-1-2 were specifically expressed in cardiac and skeletal muscle precursor cells and that miR-1 regulates ventricular cardio myocytes [51]. Following this finding, Cheng et al. reported that miRNA-1 promotes myogenesis, while miR-133 which is clustered with miR-1, stimulates myoblast proliferation [51]. Later on, Rooij et al. reported the association of miRNAs with HF and cardiac hypertrophy [52]. Additionally, more than 100 miRNAs were identified to be dysregulated during cardiac hypertrophy and HF using miRNA microarray analysis [53].

### 3.1. ncRNAs and HTN

New drug development is based on targeting genes and their encoded proteins being involved in various signalling pathways. Although pharmacotherapies using various classes of drugs have been shown to have some efficacy in reducing cardiovascular mortality (33%), major adverse cardiovascular events (29%) and HF (37%), HTN remains one of the world’s greatest public health hazards [54]. This highlights the need to further understand the underlying molecular mechanisms of HTN and targeted therapeutic treatments.

Pathogenesis of HTN entails a complex interaction of different organs and systems, including an over-activation (e.g. RAAS, sympathetic nervous systems, central and peripheral nervous system, including renal nerves) or under-activation (e.g. parasympathetic nervous system, renal dopamine, atrial natriuretic peptide) of systems that increase vascular reactivity and sodium retention, remodelling of blood vessels and dysfunction of vascular endothelia [55].

Since genetics plays pivotal role in the pathogenesis of HTN as well as other CVDs [56–58], unravelling genetic compartments that predispose one to high BP is of great significance. However, considering several identified HTN-related biomarkers, polymorphisms/mutations of genes only account for a very small part in predisposition to HTN [59].

ncRNAs are emerging as key players of transcription regulation in both health and disease states like HTN. They control basic functions in virtually all cell types relevant to the cardiovascular system and, thus, a direct involvement with BP regulation is highly probable [60]. Due to the growing technology of genomics, such as microarrays and next-generation sequencing (NGS), ncRNAs have increasingly gained attention in normal cellular processes, as well as in disease progression. Two major types of ncRNA, namely miRNA and lncRNA, have been extensively studied in both hypertensive patients and animal models [61]. One important aspect of the transcriptional regulation by ncRNAs is the fact that each individual miRNA regulates a comprehensive set of genes, thus affecting cellular pathways and governing biological functions. To date, miRNAs are the most well-characterized and widely studied group of short ncRNAs in HTN. lncRNAs are also found to play important roles in HTN. Evidences have shown that due to the characteristics of ncRNAs in affecting various processes in HTN, these molecules could be potentially considered as both biomarkers and therapeutic targets in HTN [62,63].

HTN is also regulated by epigenetic phenomena like histone modification, DNA methylation, chromatin remodelling, and especially, ncRNA-mediated targeting of various genes, e.g. G-protein-coupled receptor kinase type 4 (GRK4) phosphorylates histone deacetylase type 1 (HDAC1), promoting its nuclear export to the cytoplasm, resulting in increased Ang II receptor type 1 (AT1R) expression and greater pressure response to Ang II [64].

#### 3.1.1. miRNAs and HTN

miRNAs are master gene regulators which control the expressions of specific genes by inducing mRNA cleavage or reducing translation to regulate gene expression. A single miRNA regulates one to several hundred genes, and a single gene could be regulated by more than one miRNA. They are post-transcriptional regulators of expression in eukaryotics and are involved in many biological processes, thus having a potentially profound impact on illnesses such as HTN [65,66].

Like mRNAs, certain miRNAs are highly expressed in healthy cardiac tissue and thus probably play a role in the maintenance of normal cardiac function. Recently, some miRNAs have been found to play as biomarkers for some CVDs. It is demonstrated that specific miRNAs (miR-126, miR-155, 296–5p, let-7e and miR-181/a) are differentially expressed during the development of HTN, although the expression levels of these miRNAs are not consistent among different reports [67]. Microarray studies have found three up-regulated miRNAs (miR-425, miR-505 and miR-210) in plasma from hypertensive patients. Among these three miRNAs, plasma miR-505 was reported to be consistently elevated in all hypertensive patients [68].

Abnormal expression of miRNAs in tissues and body fluids has also been observed. It was found that serum miR-29/a level in patients with hypertensive left ventricular hypertrophy is significantly higher than that in patients with HTN alone, which suggests that miR-29/a may be directly related to the occurrence of cardiac hypertrophy in hypertensive patients [69].

Circulating miRNAs originate from tissues and are stable in serum, urine, plasma and blood cells and are resistant to endogenous RNases. Additionally, their expressions are associated with HTN. All together makes miRNAs as potential biomarkers for HTN [70].

miRNAs linked to HTN are listed in Table 1.

**Table 1.** miRNA associated with hypertension

miRNA	Strain/organism/tissue/cell type/disease	Up/Down Regulated	Method of Detection	Regulatory Role	Ref
miR-1	HASMCs/ VSMC/SHRs	Up-regulation	RT-qPCR and western blot analysis	MiR-1 regulates the proliferation of VSMCs by targeting IGF-1	[107]
let-7 g	Human PASMCs and mouse lungs induced by hypoxia	Down-regulation	qPCR-array	let-7 g and LOX-1 inhibit their expression mutually	[108]
miR-21	LEAOD, MCT, hypoxia, hypoxia/Sugen5416, lung and serum of PH patients	Down-regulation	RT-qPCR	MiR-21 lowers blood pressure in spontaneous hypertensive rats	[109]
miR-153	SHR/ Mas/ MCAs/ NT MAs	Up-regulation	RT-qPCR	MiR-153 targeting of KCNQ4 contributes to vascular dysfunction in hypertension	[110]
miR-199a-5p	HPASMCs/ HPAECs	Up-regulation	Bioinformatics tools	MiR-199a-5p influences pulmonary artery hypertension via downregulating Smad3	[111]
miR34b	VSMCs	Down-regulation	RT-qPCR	Down-regulated miR-34b is responsible for the elevation of blood pressure	[112]
miR-125a	PAH/ CTEPH	Up-regulation	RT-qPCR	MiR-125a promotes the proliferative phenotype of endothelial cells in pulmonary hypertension	[113]
miR-98	PAECs from PH patients, PAECs under hypoxia and in lung from mice induced by Sugen5416/hypoxia	Down-regulation	Bioinformatics approach using multiple prediction algorithms (miRBase, PicTar, and TargetScan)	PPARγ regulates miR-98 to modulate ET-1 expression and PAEC proliferation	[114]
miR-210	Ovine Uterine Arteries, PAECs induced by hypoxia	Up-regulation	RT-qPCR	MiR-210 modulates the hypoxic human PASMCs by MKP-1	[115]
miR-30c	PAECs from PH patients/ Rat induced by hypoxia and pulmonary arteries (PA) from PH patients	Down-regulation	qPCR-array	MiR-30c contributes to the development of hypoxia pulmonary hypertension	[116]

#### 3.1.2. lncRNAs and HTN

lncRNA is defined as a class of transcripts with more than 200 nucleotides in length that are loosely defined as RNA molecules without the protein-coding function which is now reported to have strong epigenetic regulation potentials [71]. lncRNAs have emerged as a new aspect of biology with evidences revealing their prominent roles in regulation of splicing, imprinting, epigenetic and gene transcription [72].

According to the relative genomic location on the coding region, lncRNAs are categorized in to different groups including intergenic lncRNAs (or lincRNAs), intronic lncRNAs, sense lncRNAs and antisense lncRNAs [73]. Like miRNAs, lncRNAs have been detected in the blood of HTN patients, suggesting their potentials as circulating biomarker for HTN [74].

Despite growing appreciation of the importance of lncRNAs in normal physiological processes as well as in diseases, the number of articles related to lncRNAs in HTN are limited. Although, the molecular mechanisms of lncRNAs are not fully understood and had not yet been completely decoded, the role of lncRNAs in HTN has been documented. It has been shown that dysregulation of lncRNA contributes to the pathogenesis of HTN by directly acting on vascular cells and indirectly affecting other organs [74].

An in vitro study on one particular lncRNA, XR007793, is suggestive of its up-regulation in vascular smooth muscle cells (VSMCs). Reciprocally, knocking down of XR007793 has shown to attenuate VSMC proliferation and migration. Absence of XR007793 also inhibited interferon regulatory factor 7 (irf7), signal transducers and activators of transcription 2 (stat2) and LIM only domain 2 (limo2) [75]. Results of another experiment indicated that miR-23b inhibitor reduced the expression of VSMC marker and promoted proliferation and migration of VSMC. This study showed that XR007793 aggravates the loss of function of VSMCs by negatively regulating miR-23b [76].

Several BP-related loci have been located on human genome using GWAS, including the lncRNA H19 locus which is linked to variations in systolic BP. Moreover, four SNPs (rs10757274, rs2383207, rs10757278, and rs1333049), that are located on the lncRNA, CDKN2B-AS1, were found to confer increased susceptibility to the development of HTN [77].

A recently discovered lncRNA, AK098656, a human-specific expressed lncRNA, has been verified to be upregulated while lncRNA-AK125261 was downregulated in the plasma of hypertensive patients, suggesting a promising future of lncRNA-based biomarkers in HTN [78] [78]. Plasma lncRNA growth arrest-specific 5 (GAS5) has also been reported to be decreased in patients with coronary artery disease (CAD) [79].

lncRNAs that have been found to be associated with essential HTN are summarized in Table 2.

**Table 2.** lncRNA associated with hypertension

lnRNA	Strain/organism/tissue/cell type/disease	Up/Down Regulated	Method of Detection	Regulatory Role	Ref
GAS5	VSMC/ EC	Down-regulation	RT-qPCR	GAS5 expression down regulated in HT. knockdown increased SBP and DBP and mean arterial BP (in SHR) retinal neovascularization and capillary leakage, endothelial activation and proliferation	[79]
sONE	BHRs	Down-regulation	qPCR	Lycium Barbarum L. ameliorated HTN, reduced sONE expression and improved eNOS expression compared to high salt diet rats	[103]
749 lncRNAs	SHRs/ normotensive Wistar-Kyoto (WKY) rats	Differential expression between SHR and normotensive rats	Microarray, RNASeq, mRNA trasncrptome analysis	Asb3, Chac2, Pex11b, Sp5	[117]
MALAT1	HUVECs	Up-regulation	RNASeq, Microarray, qPCR	Vessel growth, endothelial cell function	[118]
CDKN2B-AS1	HT patients/ VSMC	Up-regulation	qPCR to test if published 9p21.3 SNPs are associated with BP	Significant difference in genotype freq of the 4 SNPs betw HT and NT. Association betw rs10757274 & rs2383207 (AA) and SBP.	[119]
XR007793	Sprague-Dawley rats/ VSMCs	Up-regulation	qRT-PCR	Kncockdown of XR007793 repress VSMC proliferation & migration. Reduced transcript expression of stat2, lmo2 and irf7.	[76].
H19	PASMCs/ SD rats, C57/BL6 mice/ PAH model	Up-regulation	Discovery meta-analysis, genome-wide SNP genotype	H19-let-7b-AT1R axis contributes to the pathogenesis of PAHT by stimulating PASMCs proliferation	[120]
AK098656	Human plasma/HASMCs	Up-regulation	Microarray, qRT-PCR	Promotes VSMCs proliferation and migration	[77]

## 4. Modulating effects of polyphenols on main ncRNAs involved in HTN

**Figure 3.**
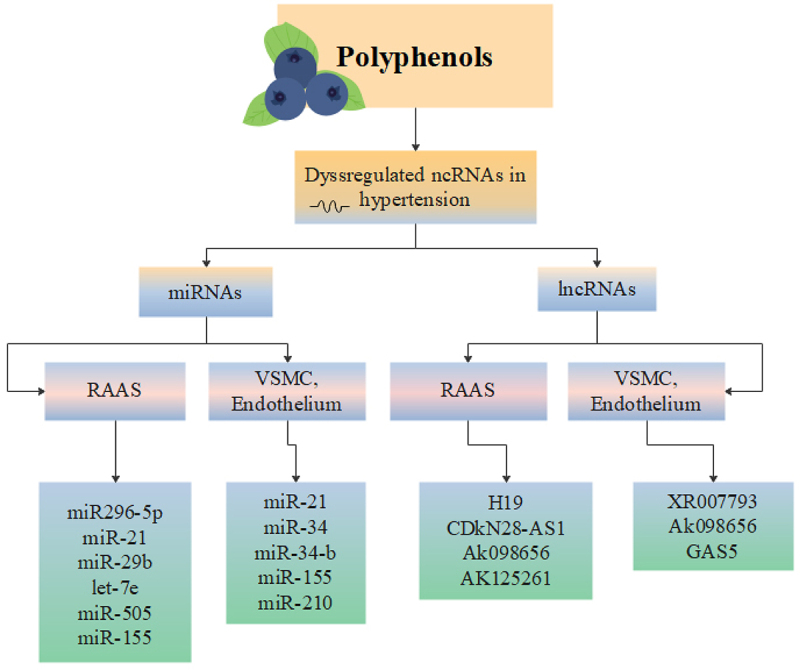
Schematic view of regulation of miRNAs and lncRNAs by polyphenols which have been dysregulated in HTN.

Many documents have reported that polyphenols provide protective effects in oxidative-related disease such as HTN [80]. Polyphenols are mainly derived from fruits, vegetables, tea, coffee, cocoa, mushrooms, beverages, and traditional medicinal herbs such as Salvia miltiorrhiza and serve as natural antioxidants [81]. Polyphenols exert their antihypertensive properties mainly [82] by modulating important signalling pathways involved in numerous cellular functions such as AKT/PI3K, NF-KB and ERK signalling pathways [83]. As described earlier, polyphenols have also direct and indirect anti-oxidant effects [84], enabling cells to reduce the damages caused by ROS [85]. Apart from the mentioned effects, three major epigenetic changes including alteration in chromatin structure, DNA methylation and most importantly expression of miRNAs, have been observed in myocytes treated by polyphenols [86]. Schematic view of regulation of miRNAs and lncRNAs by polyphenols which have been dysregulated in HTN is depicted in Fig. 3.

lncRNAs and miRNAs regulating the function of RAAS, vascular smooth muscle cells (VSMCs) and the endothelium are depicted. miRNA: microRNA; lncRNA: long non-coding RNA; RAAS: Renin angiotensin aldosterone system; VSMC: vascular smooth muscle cell.

### 4.1. Polyphenols and miRNAs

Recent findings revealed that polyphenols could interact with cellular signalling cascades, regulate the activity of transcription factors and consequently affect the expression of genes [87]. Polyphenols have been shown to affect the expression of miRNAs as well. Furthermore, expression of miRNAs can be affected by different external stimuli including nutrients such as vitamins, lipids, and phytochemicals [88]. Thus, miRNAs appear as interesting mediators in regulating polyphenols’ biological effects [88].

Flavanones are exclusively abundant in citrus fruits and present potential protective effects in cardiovascular illnesses. To date, only one study has reported the impact of two major flavanones, Hesperidin and Naringenin, on miRNA expression in Apo lipoprotein E-deficient (ApoE) mice [88]. This study revealed that Hesperidin and Naringenin, given as supplements for 2 weeks, affected the expression of 97 and 69 miRNAs respectively, with 31 miRNAs in common. These data suggest that miRNAs could be also potential targets of flavanones underlying their health effects [88].

miR-29 family is an important endogenous regulator which is proven to be linked to cardiovascular injury. Over-expression of miR-29/b can improve cardiac function. Reduction in the expression of miR-29/b has shown to decrease BP and improve the cardiac function in hypertensive rats [89].

Notably, among the polyphenols, epigallocatechin gallate (EGCG) and resveratrol have shown to modulate several classes of miRNA that are involved in all the stages of HTN development [90].

A report by Ming-Ju et al. demonstrated differentially expressed genes and miRNAs in response to EGCG treatment in pulmonary hypertension fibroblasts (PH-Fibs) using a NGS and bioinformatics approach. These changes in gene expression mainly affected oxidative and inflammatory processes, suggesting that EGCG may modulate cell signalling pathways involved in the mentioned processes, such as MAPK, NF-κB, and AMPK pathways. Moreover, it may also modulate epigenetic changes, such as DNA methylation and histone acetylation [90]. Some *in vitro* and *in vivo* studies have shown the effects of EGCG on fibroblasts, such as attenuating cell proliferation, enhancing antioxidant defence systems, and inhibiting inflammation. EGCG significantly upregulated miR-29/b-2-5, a miRNA involved in HTN. Conversely, polyphenol-rich green tea extract improved cardiomyocytes adipose tissue metabolism by down-regulating miR-29 [91].

João et al. found that a group of miRs are involved in the regulation of the inflammatory response, such as miR-21, miR-181b, miR-663, miR-30c2, miR-155 and miR-34a that were found to be highly altered in the group consuming the resveratrol-containing grape extract (GE-RES) for 12 months. Long-term supplementation with GE-RES has shown to down-regulate the expression of key pro-inflammatory cytokines with the involvement of inflammation-related miRs in circulating immune cells of type 2 diabetes (T2DM) hypertensive patients and support a beneficial immune-modulatory effect in diabetic patients with HTN [92].

Resveratrol has shown to modulate specific miRNAs in ischaemic hearts including miR-21, miR-20b, mir-27a and miR-9. miR-21 was shown to regulate ERK-MAP kinase signalling pathway in cardiac fibroblasts regulating cardiac structure and function [93]. Opposite to cancer cells, miR-21 expression is significantly reduced in ischaemic heart, thus restoring its expression in these conditions would be desirable [94].

Another miRNA which is the target of resveratrol is miR-27a. miR-27a is upregulated during cardiac hypertrophy [143]. Furthermore, miR-27/a controls PI3-kinase pathway that regulates cardiac hypertrophy and induces cardiac protection [61]. Resveratrol affects several signalling pathways in the heart including PI3-kinase pathway [144, 145] presumably by influencing the expression of miR-27/a.

In this context, other evidences indicated that resveratrol prevented MCT-induced pulmonary vascular remodelling via miR-638 regulating NR4A3/cyclin D1 pathway [95]. Another report indicated that miRNA-150-5p might be involved in the antihypertensive effect of EGCG through specificity protein 1 (SP1)/AT1R pathway in aorta of SHRs [96].

Quercetin with antioxidant and anti-proliferative properties has been reported to improved pulmonary artery hypertension (PAH) and pulmonary arterial pressure (PAP) possibly by affecting the expression of poly [ADP-ribose] polymerase 1 (PARP1) and miR-204 and their downstream targets, Hypoxia-inducible-factor 1A (HIF1a) and nuclear factor of activated T cells 2 (NFATc2) [97].

Similarly, miR-155 has been demonstrated to be related to HTN through targeting RAAS [9]. Protection from atherosclerosis and HTN is a generalized effect of polyphenols [98]. However, further studies on other polyphenols are necessary to confirm that miR-155 serves as a real target of polyphenols.

Modulation of miRs by polyphenols has also been studied in monocytes and macrophages. Polyphenols including resveratrol, quercetin, and isorhamnetin that were studied in macrophages, all target miR-155 [87]. miR-155 is proposed as a biomarker of HTN [99]. In contrast, in a monocyte cell line, THP-1, resveratrol upregulated miR-663 and impairs the up-regulation of the pro-inflammatory miR, miR-155. Interestingly, resveratrol also modulated heart and skeletal muscle functions through miRs, such as miR-20b, miR-149, miR-133, miR-21, and miR-27b [100].

Anti-inflammatory properties of quercetin and isorhamnetin were accompanied by an increase in haem oxygenase 1 levels, a downstream target of the transcription factor Nrf2, known to suppress chronic inflammation. Furthermore, pro-inflammatory miRNA-155 was down-regulated by quercetin and isorhamnetin but not by quercetin-3-glucoside [101].

### 4.2. Polyphenols and lncRNAs

lncRNAs are usually expressed in a cell-type and tissue-specific manner, indicating their importance in developmental processes and disease mechanisms [102]. The results of a study by Zhang, X., et al. showed that Lycium barbarum had anti-hypertensive effects and might lower BP by suppressing the expression of lncRNA sONE in BHR model [103]. Another study demonstrated that overexpression of H19 improved human microvascular endothelial cells (HMEC-1) viability, migration and tube formation capacity, showing pro-angiogenic effects. Alongside, overexpression of H19 leads to down-regulation of miR-181a which, in turn, activates JNK and AMPK signalling and ultimately promotes neovascularization. These findings have provided *in vitro* evidence that H19 might be one of the potential targets for the treatment of atherosclerosis-related diseases, such as peripheral artery disease (PAD) [104].

Fisetin, a flavonoid in found in fruits, nuts and vegetables possesses antioxidant and anti-inflammatory properties. Fisetin effectively suppresses calcineurin-NFATC3 involved cellular hypertrophic effects as observed in H9c2 cells and in SHR hearts. Fisetin also enhances cardiac function and restores cardiac morphology and therefore, may be considered as a potential agent against HTN associated hypertrophy and cardiac deterioration [105]. Protective effect of fisetin against Ang II–induced apoptosis by Activation of IGF-IR-PI3K-Akt Signalling in SHR potentially restores cardiac function [83].

Overall, the antihypertensive mechanisms of polyphenols have revealed that these natural-based molecules could target the expression of main lncRNAs.

## 5. Challenges and outlook

Application of polyphenols may be considered as a new therapeutic strategy for HTN [106]. Accordingly, future studies should focus on:

(a) Considering the promising results of various animal models, ncRNAs could be considered as novel therapeutic options as either synthetic ‘agomiRs’ or ‘antagomiRs’.

(b) Studying the anti-inflammatory effects of polyphenols and their analogues focusing on other types of ncRNAs.

(c) Demonstration of the safety and effectiveness of polyphenols in clinical trials.

## 6. Conclusion

In spite of the continuous efforts of the scientific community to understand the pathogenesis of HTN, many aspects at the molecular level remain elusive. The elucidation of molecular processes regulated by (small) RNA sequencing technologies and the identification of novel ncRNA targets in the pathogenesis of HTN are valuable and exciting strategies that may eventually lead to the development of novel treatments to prevent and reverse the consequences of HTN.

There are currently a small number of studies that support the role of ncRNAs in BP regulation and HTN. ncRNAs are, however, a relatively new area of investigation in HTN research.

Various functions of ncRNAs, in particular, miRNAs and lncRNAs, have unlocked opportunities and developments in clinical trials for RNA interference (RNAi) as the next venue for medical therapy. New strategies in drug development could emphasize on repressing/inhibiting the upregulated ncRNAs. Alternatively, deficient ncRNAs could be replaced or enhanced by the overexpression of ncRNAs or utilizing synthetic ncRNAs. The advantages that ncRNAs provide over the conventional drug molecules are their action on any gene of interest and their potential of gene repression, which some traditional drug molecules could not access. Application of ncRNAs in CVDs especially HTN may open up a new venue for smart treatment of such illnesses.
